# Community Trial on Heat Related-Illness Prevention Behaviors and Knowledge for the Elderly

**DOI:** 10.3390/ijerph120303188

**Published:** 2015-03-17

**Authors:** Noriko Takahashi, Rieko Nakao, Kayo Ueda, Masaji Ono, Masahide Kondo, Yasushi Honda, Masahiro Hashizume

**Affiliations:** 1 Department of Paediatric Infectious Diseases, Institute of Tropical Medicine, Nagasaki University, 1-12-4 Sakamoto, Nagasaki 852-8523, Japan; E-Mail: pediatric.nagasaki@gmail.com; 2 Graduate School of Biomedical Sciences, Nagasaki University, 1-12-4 Sakamoto, Nagasaki 852-8523, Japan; 3 National Center for Child Health and Development, 2-10-1 Okura, Setagaya, Tokyo 157-8535, Japan; 4 Department of Nursing, Graduate School of Biomedical Science, Nagasaki University, 1-7-1 Sakamoto, Nagasaki 852-8520, Japan; E-Mail: rieko@nagasaki-u.ac.jp; 5 The National Institute for Environmental Studies, 16-2 Onogawa, Tsukuba, Ibaraki 305-8506, Japan; E-Mails: ueda.kayo.8x@kyoto-u.ac.jp (K.U.); onomasaj@nies.go.jp (M.O.); 6 Faculty of Medicine, University of Tsukuba, 1-1-1 Tennodai, Tsukuba, Ibaraki 305-8575, Japan; E-Mail: mkondo@md.tsukuba.ac.jp; 7 Faculty of Health and Sport Sciences, University of Tsukuba, 1-1-1 Tennodai, Tsukuba, Ibaraki 305-8575, Japan; E-Mail: honda@taiiku.tsukuba.ac.jp

**Keywords:** heat-related illness, heat health warning, behavior and knowledge change, elderly people, community trial

## Abstract

This study aims to explore whether broadcasting heat health warnings (HHWs), to every household and whether the additional home delivery of bottled water labeled with messages will be effective in improving the behaviors and knowledge of elderly people to prevent heat-related illness. A community trial on heat-related-illness-prevention behaviors and knowledge for people aged between 65 and 84 years was conducted in Nagasaki, Japan. Five hundred eight subjects were selected randomly from three groups: heat health warning (HHW), HHW and water delivery (HHW+W), and control groups. Baseline and follow-up questionnaires were conducted in June and September 2012, respectively. Of the 1524 selected subjects, the 1072 that completed both questionnaires were analyzed. The HHW+W group showed improvements in nighttime AC use (*p* = 0.047), water intake (*p* = 0.003), cooling body (*p =* 0.002) and reduced activities in heat (*p* = 0.047) compared with the control, while the HHW group improved hat or parasol use (*p* = 0.008). An additional effect of household water delivery was observed in water intake (*p* = 0.067) and cooling body (*p* = 0.095) behaviors. HHW and household bottled water delivery improved heat-related-illness-prevention behaviors. The results indicate that home water delivery in addition to a HHW may be needed to raise awareness of the elderly.

## 1. Introduction

Heat wave events are becoming a serious public health concern. The heat wave that occurred in Western Europe in 2003 was estimated to have led to 14,800 excess deaths in France [1] and 71,000 in 16 European countries, including France [2]. The 2009 heat wave in Victoria, Australia, was reported to have caused 347 excess deaths [3]. In Chicago, USA, during the 1995 heat wave, there were 1072 excess hospital admissions among all age groups and 838 among people aged 65 and older [4]. The elderly are more vulnerable to heat because of changes in their thermoregulatory system [5]. In Japan, it has been reported that about half the patients taken to the hospital by ambulance due to heat stroke were elderly people over the age of 65 years [6,7]. To reduce the adverse health effects of hot weather, heat health warning systems (HHWSs) that include early alerts and emergency measures in response to forecasts of weather conditions that violate predetermined trigger levels have been introduced in cities around the world [8], and a HHWS has been operated in Japan since 2006 [9]. Although some studies reported the effectiveness of HHWSs to reduce excess deaths, most studies simply compared the number of deaths during a hot period where no HHWS was implemented with a similar hot period after a HHWS was implemented, without including a control (non-intervention) group [10]. In such studies, it is hard to establish a robust causal relationship between the implementation of a HHWS and reduced mortality. Also, in these “natural” intervention studies, there was no evidence of whether the warnings reached and were heeded by the target individuals, especially the elderly who are more likely to have difficulty in accessing such information. Because mere availability of a HHWS does not necessarily lead to behavioral changes to take protective actions [10], and because awareness and perception of themselves as vulnerable are more likely to trigger protective actions [11,12], individual-based approaches to raise the awareness of at risk individuals may also be needed.

Here, we aim to explore whether broadcasting heat health warnings (HHWs) to every household using existing optical networks in the community, and whether the additional home delivery of bottled water labeled with messages about the prevention of heat-related illness (individual-based approach) will be effective in improving the behaviors and knowledge of elderly people to prevent heat-related illness.

## 2. Materials and Method

### 2.1. Study Design

This study was a community trial with three arms: (1) dissemination of HHW to each household (HHW group); (2) dissemination of HHW plus bottled water delivery to each household (HHW+W group); and (3) no-intervention (control) group. The intervention period was 9 weeks in the summer of 2012 and the data were collected pre- and post-intervention.

### 2.2. Settings and Participants

**Figure 1:**
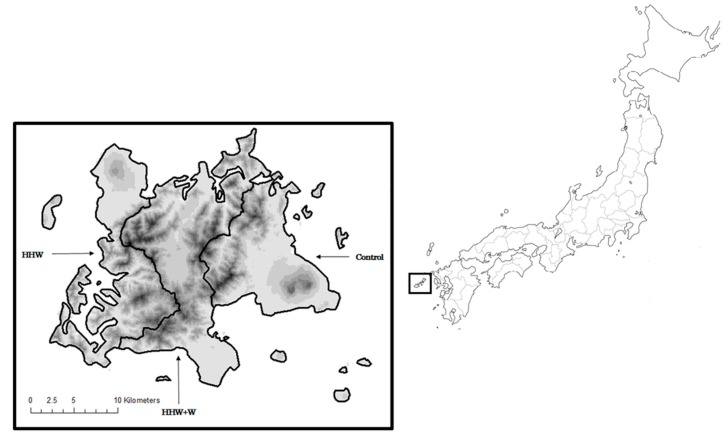
The map of the study site.

The study was conducted in Goto on Fukue island (population 37,875, area 158.45 km^2^), one of the remote islands in Nagasaki prefecture, Japan (Figure 1), where 32% of the population were aged 65 years and older. Among the five administrative areas of Fukue island, two areas were assigned to the HHW group, two were assigned to the HHW+W group, and one, the most populous area where no audio terminals or optical networks were installed in any of the households, was assigned to the control group. People aged between 65 and 84 residing in the five areas on 30 April 2012 were eligible for inclusion in the study. Because people aged 85 and older have a high possibility of suffering from dementia or cognitive disorders, they were excluded from the study. Stratified random sampling in each arm was conducted to select 127 people in each age group (65–74 and 75–84 year-olds) and for each sex, giving a total of 508 people in each arm. Random sampling was performed by the Department of Statistics of Goto City based on the Basic Resident Register.

### 2.3. Sample Size

Assuming that the use of air conditioning (AC) would increase from 75 to 85% in the intervention group at a 5% significance level with 80% power, the overall sample size required was 810 (270 in each arm of the study). Accounting for a 20% loss to follow up, a 20% non-response rate, and a design effect (1.2), the final sample size required was estimated to be 1524 (508 in each arm).

### 2.4. Intervention

HHWs were delivered for 9 weeks (from 1 July to 1 September 2012) to each household in the HHW and HHW+W groups from the city hall through existing audio terminals connected to an optical network. The audio terminals and the optical network were installed by the local government in 2008 to disseminate disaster prevention information, and they were present in each household in the administrative areas of HHW and HHW+W groups. HHWs were broadcasted when the following weather conditions were met: the predicted wet-bulb globe temperature (WBGT) was 28 °C or higher, and the predicted ambient temperature was 31 °C or higher. The predicted WBGT was provided by e-mail from the National Institute of Environmental Studies to the city hall at around 6 am, and the predicted ambient temperature (updated at 5 am and 11 am) was obtained from the Japan Meteorological Agency website [13]. Municipal staff confirmed the up-to-date temperatures and decided whether they would deliver a HHW in the mornings (10 am) and afternoons (1 pm).

In addition to the HHW delivery, two 500 mL bottles of water with short messages about heat-related illness prevention behaviors were delivered by couriers to each household in the HHW+W group once a week for 5 weeks during the intervention period. The idea was to remind the people in the group to drink water in hot weather, and the messages also recommended drinking tap water after finishing the two bottles of water. For individuals who had restricted water intake recommended by a medical doctor, we advised them to follow their doctor’s recommendation.

Pamphlets created by the Ministry of the Environment (Japan) about heat-related-illness prevention were delivered to the two intervention groups when the baseline questionnaires were collected. Chilling pads were also distributed to participants in the HHW+W group as a reward for answering the questionnaires. Chilling pads are towel-like shaped products that were developed to prevent heat stroke. Once chilling pads are soaked in water, cold sense can be gained for a certain period of time.

### 2.5. Data Collection

Letters of consent and the baseline questionnaires were sent to all the selected people by post before the end of June 2012. Follow-up questionnaires, with the same multiple-choice questions, were sent to respondents by post at the end of August after the 9-week intervention period. The questionnaires were collected by 121 local welfare commissioners (*Minsei-iin*) in Goto. *Minsei-iins* are usually assigned by each municipality in accordance with the law. Explanatory briefings about data collection were given in June 2012, and follow-up sessions were also implemented in the following 3 months. The *Minsei-iin* collected the baseline questionnaire and letters of consent at the end of June and the follow-up questionnaire in September 2012. When *Minsei-iin* reported that a selected participant was suffering from dementia or cognitive disorders and had difficulty in answering the questionnaire, that individual was excluded from the study. If a participant had a writing disability, the *Minsei-iin* helped them fill in the answers.

### 2.6. Outcome Measures

The questionnaires consisted of three parts: information about the participants’ demography and lifestyle; behavior modification during heat; and knowledge about heat-related illnesses. For behavior modification, 13 outcomes were defined; six were related to the use of AC and electric fans (EFs), and seven were related to the actions of the participants to prevent heat-related illness. Questions about cooling devices related to operating times of AC and/or EFs (daytime and nighttime), at what temperature the AC was switched on, and how the EF was used. Questions about awareness or steps to prevent heat-related illness included frequency of alcohol intake, water intake, cooling of the body, taking a rest, reduced activities during daytime, type of clothing, and use of hats or parasols outside. The details of the questions are available in the Appendix. For knowledge of heat-related illness, there were 25 questions, each with two options: yes or no. The questions related to prevention, symptoms, basic information of heat-related illness, perspiration, and effective use of EFs. We asked the participants to answer the baseline questionnaire to help us determine the trends in participant use of cooling devices and attitudes or actions during the summer in 2011. In the follow-up questionnaire, we asked the participants to answer based on how they responded to the heat during the 2012 summer.

### 2.7. Statistical Analysis

Based on their responses to the baseline and follow-up questionnaires, the participants were divided into improved or non-improved individuals for behavioral modifications. The definitions used in this study for the improvement for each variable are given in the Appendix. Briefly, for the question about the length of AC use, for example, the participants who reported longer AC operation times in the follow-up survey compared with in the baseline survey were categorized as improved, the others as non-improved. The odds ratios (ORs) and 95% confidence intervals (95% CIs) for the difference in improvement rates between the three groups were estimated using multivariable logistic regression analysis adjusted for individual characteristics and lifestyle (sex, age, education, family structure, employment, community involvement, frequency of listening to the radio, and residential type). For knowledge about heat-related illness, the differences in the mean number of correct answers were compared between the three groups using the Mann-Whitney *U* test. Stata software version 12 (Stata Corp, College Station, TX, USA) was used for the statistical analysis. *p* < 0.05 was considered as evidence, and 0.05 < *p* < 0.10 as suggestive.

### 2.8. Ethics

All subjects gave their informed consent for inclusion before they participated in the study. The study was conducted in accordance with the Declaration of Helsinki, and the protocol was approved by the Ethics Committee of the Institute of Tropical Medicine, Nagasaki University.

## 3. Results

### 3.1. Respondents

**Figure 2:**
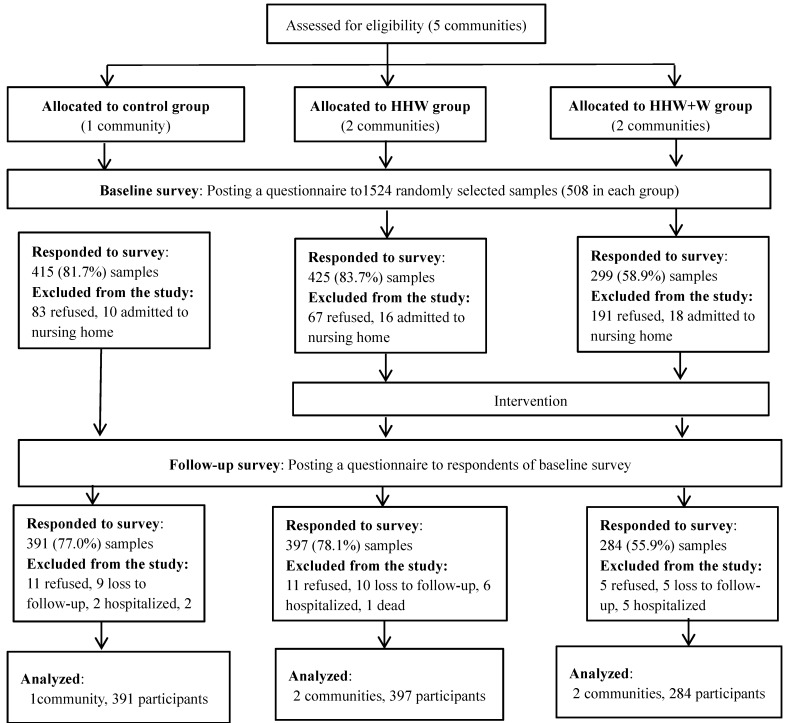
The flow of the participants through the trial.

Of the 1524 elderly people approached, 44 (2.9%) were in a nursing home and were excluded from the study. A further 341 (22.4%) declined to participate in the study because of a lack of interest or precarious health. A total of 1139 returned their letters of consent and the baseline questionnaire. Of these, 67 dropped out for various reasons (Figure 2), leaving 1072 (70.3%) participants who completed the study. Moreover, we showed the consort checklist in Table A1. Table 1 shows the baseline characteristics of the participants in the three study groups. Each item in Table 1 includes missing data due to non-respondent and the missing data was handled as missing. Participants in the control group had a higher educational status than those in the other groups, and tended to live in a flat or a building made from reinforced concrete. However, there were no other significant differences in the personal characteristics and lifestyle among the groups. Most participants had their own AC or EFs at home and only two participants owned neither.

**Table 1:** Demographic and socio-economic characteristics of the participants as reported in the baseline questionnaire.

Participants Characteristics		Control (n = 391)	HHW (n = 397)	HHW+W (n = 284)	*p*-Value
		n (%)	n (%)	n (%)	
Age	Mean (SD)	74.3 (5.7)	74.3 (5.5)	73.9 (5.3)	0.276
	65–74	190 (48.6)	198 (49.9)	156 (54.9)	
	75–84	193 (49.4)	181 (45.6)	123 (43.3)	
Sex	Male	194 (49.6)	192 (48.4)	139 (48.9)	0.900
	Female	194 (49.6)	199 (50.1)	142 (50.0)	
Education	Junior high school	190 (48.6)	254 (64.0)	189 (66.6)	<0.001
	High school	117 (29.9)	71(17.9)	46 (16.2)	
	College/University	38 (9.7)	24 (6.1)	13 (4.6)	
Employment	Employed	131 (33.5)	123 (31.0)	96 (33.8)	0.154
	Unemployed	253 (64.7)	254 (64.0)	178 (62.7)	
Community involvement	Participate	111 (28.4)	115 (29.0)	82 (28.9)	0.596
	Do not participate	266 (68.0)	258 (65.0)	188 (66.2)	
Family structure	Living alnoe	95 (24.3)	94 (23.7)	68 (23.9)	0.964
	Living together	291(74.4)	299 (75.3)	214 (75.4)	
Regular medical treatment	Receive	120 (30.7)	91 (22.9)	73 (25.7)	0.125
	Do not receive	261 (66.8)	299 (75.3)	205 (72.2)	
Residence type	House	361 (92.3)	386 (97.2)	278 (97.9)	0.00
	Flat	14 (3.6)	9 (2.3)	2 (0.7)	
	Other	10 (2.6)	0 (0.0)	1 (0.4)	
Residencial structure	Wooden house	346 (88.5)	379 (95.5)	271 (95.4)	0.00
	Reinforced concrete	38 (9.7)	15 (3.8)	11 (3.9)	
TV ownership	Own	379 (96.9)	384 (96.5)	279 (98.2)	0.495
	Do not own	2 (0.5)	5 (1.3)	1 (0.4)	
Internet usage	Use	39 (10.0)	21 (5.3)	14 (4.9)	0.007
	Do not use	342 (87.5)	353 (88.9)	254 (89.4)	
Radio usage	Frequent	105 (28.2)	42 (11.3)	69 (25.9)	<0.001
	Infrequent (up to 2 times / week)	268 (71.9)	329 (88.7)	197 (74.1)	
Newspaper	Subscribe	232 (59.3)	170 (42.8)	130 (45.8)	<0.001
	Do not subscribe	148 (37.9)	217 (54.7)	149 (52.5)	
Alcohol intake	Drink	129 (33.0)	108 (27.2)	93 (32.8)	0.322
	Do not drink	256 (65.5)	279 (70.3)	184 (64.8)	
Smoking status	Smoke	46 (11.8)	41 (10.3)	25 (8.80)	0.077
	Used to smoke	60 (15.4)	42 (10.6)	31 (10.9)	
	Have never smoked	275 (70.3)	293 (73.8)	211 (74.3)	
AC ownership	Own	358 (91.6)	351 (88.4)	264 (93.0)	0.217
	Do not own	32 (8.2)	42 (10.6)	19 (6.7)	
Fan ownership	Own	366 (93.6)	385 (97.0)	273 (96.1)	0.162
	Do not own	22 (5.6)	9 (2.3)	9 (3.2)	

Note: The total for each characteristic is not 100% because non-respondents were excluded.

### 3.2. Weather Conditions and HHWs

**Figure 3:**
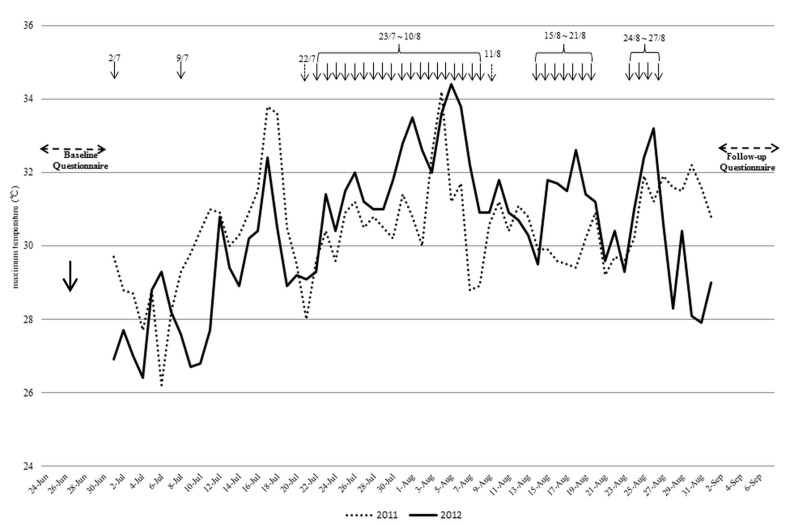
Daily maximum temperatures in the summers of 2011 and 2012 and the dates on which the HHWs were issued in the 2012 summer.

The average daily maximum temperatures in July 2011 and 2012 were 26.63 °C and 26.55 °C, respectively. This temperature difference was not statistically significant; however, the temperature difference between August 2012 (28.06 °C) and August 2011 (27.22 °C) was statistically significant (*p* < 0.01) (Figure 3). HHWs were issued for 12 days in July 2012 and for 22 days in August 2012. Most of the HHWs were issued at around 10 am, except for one day in August when the HHW was issued in the afternoon.

### 3.3. Improvement of Behaviors to Prevent Heat-Related Illness

The crude and adjusted ORs for the improvements in the use of AC and EFs in the three groups are shown in Table 2. For the HHW+W group, both the crude and adjusted ORs for the operating times of nighttime AC were 1.49, which was significantly higher than in the control group. However, there was no evidence of the improvement in the daytime AC use, temperature to turn on AC ≤ 27 °C, room temperature setting of AC ≤ 27 °C, EF use or simultaneous use of AC and EFs (under “effective use of EF” in Table 2) in either of the intervention groups.

There was evidence of an improvement in the frequency of water intake and cooling body in the HHW+W group compared with in the control group (*p* = 0.003 and *p =* 0.002*,* respectively) and also compared with in the HHW group (*p* = 0.067 and *p* = 0.095, respectively). An improvement in the frequency of taking a break (*p* = 0.088), reduced activities in the heat (*p* = 0.093), and increase in hat or parasol use (*p* = 0.008) was also found in the HHW group compared with in the control.

**Table 2:** Odds ratios (ORs) for improved behaviors to prevent heat-related illness before/after intervention.

	Control		HHW					HHW+W				*p*-Value HHW *vs*. HHW+W
	No. of improved people (%)	No. of improved people (%)	**Crude**		**Adjusted**		No. of improved people (%)	**Crude**		**Adjusted**		
			OR (95% CI)	*p*-value	OR (95%CI)	*p*-value		OR (95% CI)	*p*-value	OR (95% CI)	*p*-value	
1. Day time AC use (N = 905)	182 (54.3)	164 (50.9)	0.87 (0.64, 1.19)	0.383	0.94 (0.65, 1.35)	0.737	129 (52.0)	0.91 (0.66, 1.27)	0.580	0.88 (0.60, 1.29)	0.505	0.733
2. Night time AC use (N = 902)	133 (39.7)	134 (41.5)	1.08 (0.79, 1.47)	0.641	1.10 (0.76, 1.60)	0.606	121 (49.6)	1.49 (1.07, 2.08)	0.018	1.49 (1.01, 2.19)	0.047	0.141
3. Temperatures to turn on AC (N = 789)	28 (9.4)	35 (12.4)	1.37(0.81, 2.31)	0.245	1.41 (0.75, 2.67)	0.289	26 (12.4)	1.37 (0.78, 2.41)	0.275	1.31 (0.68, 2.52)	0.419	0.825
4. Room temperature settings of AC (N = 831)	159 (51.3)	165 (56.1)	1.21 (0.88, 1.67)	0.234	1.37 (0.93, 2.01)	0.106	114 (50.2)	0.96 (0.68, 1.35)	0.806	1.09 (0.73, 1.63)	0.676	0.273
5. Electric fan (EF) use (N = 906)	185 (56.4)	192 (58.0)	1.07 (0.78, 1.45)	0.677	1.02 (0.71, 1.48)	0.910	138 (55.9)	0.98 (0.70, 1.36)	0.899	0.89 (0.60, 1.31)	0.553	0.487
6. Effective use of EF (N = 628)	118 (53.2)	107 (46.9)	0.78 (0.54, 1.13)	0.187	0.94 (0.60, 1.49)	0.804	79 (44.4)	0.70 (0.47, 1.05)	0.082	0.72 (0.45, 1.16)	0.176	0.270
7. Frequency of alcohol intake (N = 952)	224 (65.1)	247 (70.4)	1.27 (0.92, 1.75)	0.139	1.34 (0.89, 2.02)	0.158	161 (62.7)	0.90 (0.64, 1.26)	0.532	1.02 (0.67, 1.57)	0.921	0.225
	No. of improved people (%)	No. of improved people (%)	**Crude**		**Adjusted**		**Crude**	**Crude**		**Adjusted**		
			OR (95% CI)	*p*-value	OR (95%CI)	*p*-value		OR (95% CI)	*p*-value	OR (95% CI)	*p*-value	
8. Clothing type (N = 986)	259 (71.6)	248 (68.1)	0.85 (0.62, 1.17)	0.316	0.95 (0.64, 1.40)	0.781	182 (70.0)	0.93 (0.65, 1.32)	0.675	1.11 (0.73, 1.70)	0.629	0.460
9. Frequency of water intake (N = 1002)	138 (37.8)	147 (39.7)	1.08(0.81, 1.46)	0.593	1.25 (0.87, 1.78)	0.224	132 (49.4)	1.61 (1.17, 2.21)	0.004	1.77 (1.21, 2.58)	0.003	0.067
10. Cooling body (N = 978)	91 (25.5)	106 (29.7)	1.23 (0.89, 1.72)	0.209	1.34 (0.91, 1.97)	0.137	101 (38.3)	1.81 (1.28, 2.55)	0.001	1.87 (1.26, 2.80)	0.002	0.095
11. Frequency of taking a break (N = 931)	108 (31.9)	115 (33.1)	1.06 (0.77, 1.45)	0.740	1.39 (0.95, 2.03)	0.088	82 (33.6)	1.08 (0.76, 1.54)	0.657	1.19 (0.79, 1.79)	0.414	0.445
12. Reduced activities in the heat (N = 961)	234 (67.2)	258 (72.3)	1.27 (0.92, 1.75)	0.146	1.40 (0.95, 2.07)	0.093	184 (71.9)	1.25 (0.88, 1.77)	0.223	1.54 (1.01, 2.37)	0.047	0.656
13. Hat or parasol use (N = 1021)	282 (75.6)	317 (82.9)	1.65 (1.15, 2.37)	0.006	1.80 (1.17, 2.77)	0.008	223 (83.6)	1.56 (1.05, 2.32)	0.027	1.39 (0.88, 2.20)	0.163	0.299

Multivariable models include age, sex, education, family structure, employment, community involvement, frequency of listening radio, and residential type as covariates. The details of the criteria for improved behaviors are available in the Appendix.

**Table 3:** Changes in participant knowledge score about heat-related illness before/after intervention.

		Control			HHW			HHW+W			*p*-Value for HHW *vs*. Control ^b^	*p*-Value for HHW +W *vs.* Control ^b^	*p*-Value for HHW *vs.* HHW+W ^b^
		Baseline	Follow-up	*p*-Value ^a^	Baseline	Follow-up	*p*-Value ^a^	Baseline	Follow-up	*p*-Value ^a^			
Total knowledge score (Mean ± SD)		16.5 ± 4.5	17.2 ± 4.6	<0.001	16.1 ± 5.2	16.1 ± 5.7	0.188	16.4 ± 5.1	16.5 ± 5.7	0.064	0.057	0.163	0.698
**Question**	**Answer**	**Number of the participants who selected the correct answer, n (%)**											
1. Can usage of cooling devices prevent heat stroke?	Yes	308 (78.8)	337 (86.2)	-	299 (75.3)	322 (81.1)	-	231 (81.3)	234 (82.4)	-			
2. Can wearing thick clothes prevent heat stroke?	No	291 (74.4)	312 (79.8)	-	276 (69.5)	255 (64.2)	-	202 (71.1)	192 (67.6)	-			
3. Can staying at cool spots prevent heat stroke?	Yes	332 (84.9)	325 (83.1)	-	326 (82.1)	295 (74.3)	-	238 (83.8)	215 (75.7)	-			
4. Can cooling body down prevent heat stroke?	Yes	296 (75.7)	313 (80.1)	-	287 (72.3)	287 (72.3)	-	210 (73.9)	224 (78.9)	-			
5. Is dehydration one of the symptoms of heat stroke?	Yes	350 (89.5)	351 (89.8)	-	345 (86.9)	330 (83.1)	-	256 (90.1)	242 (85.2)	-			
6. Is tiredness one of the symptoms of heat stroke?	Yes	324 (82.9)	329 (84.1)	-	329 (82.9)	320 (80.6)	-	231 (81.3)	230 (81.0)	-			
Total knowledge score (Mean ± SD)	16.5 ± 4.5	17.2 ± 4.6	<0.001	16.1 ± 5.2	16.1 ± 5.7	0.188	16.4 ± 5.1	16.5 ± 5.7	0.064	0.057	0.163	0.698	
7. Are dizziness and light-headedness one of the symptoms of heat stroke?	Yes	353 (90.3)	351 (89.8)	-	340 (85.6)	335 (84.4)	-	248 (87.3)	246 (86.6)	-			
8. Is headache one of the symptoms of heat stroke?	Yes	296 (75.7)	323 (82.6)	-	311 (78.5)	315 (79.4)	-	225 (79.2)	234 (82.4)	-			
9. Is feeling nauseous one of the symptoms of heat stroke?	Yes	293 (74.9)	320 (81.8)	-	298 (75.3)	307 (77.3)	-	224 (78.9)	235 (82.8)	-			
10. Is reduction in appetite one of the symptoms of heat stroke?	Yes	306 (78.3)	330 (67.5)	-	315 (79.4)	321 (65.0)	-	219 (77.1)	215 (63.4)	-			
11. Is sweating one of the symptoms of heat stroke?	Yes	268 (68.5)	264 (67.5)	-	262 (66.0)	258 (65.0)	-	197 (69.4)	180 (63.4)	-			
12. Is muscle cramp one of the symptoms of heat stroke?	Yes	161 (41.2)	202 (51.7)	-	157 (39.6)	198 (49.9)	-	120 (42.3)	152 (53.5)	-			
Total knowledge score (Mean ± SD)		16.5 ± 4.5	17.2 ± 4.6	<0.001	16.1 ± 5.2	16.1 ± 5.7	0.188	16.4 ± 5.1	16.5 ± 5.7	0.064	0.057	0.163	0.698
13. Can sweating reduce body temperature?	Yes	254 (65.0)	252 (64.5)	-	217 (54.7)	217 (54.7)	-	169 (59.5)	150 (52.8)	-			
14. Can sweating negatively affect people with hypertension or cardiac diseases?	Yes	191 (48.9)	186 (47.6)	-	174 (43.8)	168 (42.3)	-	140 (49.3)	134 (47.2)	-			
15. Do people sweat when not really feeling the heat?	Yes	186 (47.6)	200 (51.2)	-	215 (54.2)	195 (49.1)	-	135 (47.5)	148 (52.1)	-			
16. Does sweating a lot make people exhausted?	Yes	303 (77.5)	319 (81.6)	-	305 (76.8)	307 (77.3)	-	222 (78.2)	220 (77.5)	-			
17. Does heat stroke always make people thirsty?	No	114 (29.2)	137 (35.0)	-	116 (29.2)	129 (32.5)	-	92 (32.4)	108 (38.0)	-			
18. Is heat stroke getting worse?	Yes	291 (74.4)	310 (79.3)	-	307 (77.3)	296 (74.6)	-	214 (75.4)	204 (71.8)	-			
Total knowledge score (Mean ± SD)		16.5 ± 4.5	17.2 ± 4.6	<0.001	16.1 ± 5.2	16.1 ± 5.7	0.188	16.4 ± 5.1	16.5 ± 5.7	0.064	0.057	0.163	0.698
19. Are people with hypertension or cardiac diseases more likely to get heat stroke?	Yes	206 (52.7)	192 (49.1)	-	196 (49.4)	189 (47.6)	-	140 (49.3)	137 (48.2)	-			
20. Does heat stroke occur in sleep?	Yes	317 (81.1)	332 (84.9)	-	315 (79.4)	323 (81.4)	-	220 (77.5)	253 (89.1)	-			
21. Are temperatures only the factor related to heat stroke?	No	284 (72.6)	305 (78.0)	-	307 (77.3)	291 (73.3)	-	210 (73.9)	201 (70.8)	-			
22. Does heat stroke occur in early summer or winter?	Yes	228 (58.3)	196 (50.1)	-	216 (54.4)	213 (53.7)	-	165 (58.1)	153 (53.9)	-			
23. Can electric fans decrease ambient temperatures?	No	128 (32.7)	135 (34.5)	-	100 (25.2)	101 (25.4)	-	71 (25.0)	77 (27.1)	-			
24. Are electric fans effective when used in conjunction with AC?	Yes	343 (87.7)	339 (86.7)	-	344 (86.7)	348 (87.7)	-	237 (83.5)	246 (86.6)	-			
25. Are fans effective to prevent heat stroke even if the humidity level is high?	No	36 (9.2)	69 (17.7)	-	27 (6.8)	65 (16.4)	-	41 (14.1)	47 (16.6)	-			

^a^ Wilcoxon tests were conducted using mean scores to compare baseline and follow-up. ^b^ Mann-Whiteney *U* tests were conducted using mean difference for comparisons among groups.

### 3.4. Knowledge of Heat-Related illness

The mean scores of knowledge about heat-related illness were improved in the control (*p* < 0.001) and in the HHW+W group (*p* = 0.064) (Table 3). Overall, participants gained a comparatively better understanding about the prevention and symptoms of heat-related illness. Knowledge about the effectiveness of EFs to reduce ambient temperature (item No.23 in Table 3) and heat stroke events (item No.25 in Table 3) was low in all three groups. There was no evidence for the improvement of the knowledge scores among the three groups.

## 4. Discussion

The aim of this study was to assess the effectiveness of household dissemination of HHWs and additional household bottled water delivery to improve the behavior and knowledge of the elderly to help prevent heat-related illness. Earlier studies have investigated the effectiveness of HHWSs simply by estimating decreased excess deaths without comparing them with non-intervention groups [10], and no evidence of whether the warning reached to the target individuals was reported. In this study, we used a study design that overcame these defects in the earlier studies. We found that HHW+W was significantly associated with improved behaviors (*i.e*. nighttime AC use, frequency of water intake, cooling of the body, and reduced activity in the heat), while HHWs alone improved taking a break, reduced activities in the heat, and encouraged hat or parasol use. An additional effect of household water delivery was observed in water intake and cooling the body.

The HHWSs in Europe tend to operate more at national levels than the HHWSs in other countries [14]. Further, in Europe, the action plans include not only HHWs but also other provisions, such as activation of hotlines, increased medical staff, and monitoring vulnerable people [14]. Although other countries and regions have implemented HHWSs at national and local levels [10], only a few municipalities have established action plans with multiple provisions for heat [15,16] and the effects of an individual-based approach for the elderly has never been assessed [10,14,15,16]. Therefore, in the present study, we defined three arms to investigate the effectiveness of a community-based approach and an additional individual-based approach in modifying behaviors in an elderly Japanese population.

The HHW+W group showed a significant improvement in nighttime AC use compared with the control, implying that, in addition to the HHWs, repeatedly sending bottled water with messages might have worked as a reminder that indirectly enhanced behaviors towards heat. Moreover, because of water delivery, the participants in the HHW+W group had chances to meet third persons (*i.e*. couriers), while no specific interposition of couriers was taken place in the other groups. Social isolation could exacerbate heat-related deaths or illness [17,18], suggesting that the interposition of a person could help prevent heat related illness in the elderly, even when the person was a non-family member. There may be concerns about a possible contamination of intervention effects in the control group by the *Minsei-iin* visits to collect baseline and follow-up questionnaires: this may have slightly affected behaviors and knowledge of the control group. However, because the *Minsei-iin* collected the questionnaires exactly in the same way in the intervention groups too, it seems unlikely that this would have introduced bias in the study results.

The improvements gained by the individual-based approach may partly be because, by distributing bottled water, there was an improvement in water intake, suggesting that the water bottle itself could have worked as a reminder to increase water intake. The distribution of chilling pads might also have improved the frequency of cooling of the body, even though they were distributed as rewards. These results suggest that the participants might have more easily understood how to prepare for heat because of the products that were distributed. Thus, product distribution could possibly be effective for the elderly as a trigger for behavior modification.

Knowledge score of heat-related illness in the control group was significantly improved, even when no interventions were conducted. Because of high TV ownership, we assume that this is because most participants could have accessed information about heat through weather news. In addition, though the proportions themselves were not high, newspaper, radio and internet use were also higher in the control group than in the intervention groups, and this may have also contributed to the improvement of the score in the control. While we incorporated radio use in the model to control for the potential confounding effects of these mass media, the intervention effects may still have been biased toward the null.

The results showed that many participants had insufficient knowledge of EFs, because most of them believed that EFs reduced ambient temperatures. Although EFs can help ventilate a room, sufficient evidence that EFs are protective against heat has not been gained so far [17,18]. EFs can increase convective heat gain when ambient temperatures are ≥ 35 °C, because hot air is blown over the body [19]; further, high humidity can weaken the ability of fans to dissipate heat through evaporative cooling. Therefore, at high temperatures and humidity, the risk of heat stroke is high when only fans are used for cooling [20], whereas AC has been proven to alleviate heat-related deaths or illness [21,22]. Thus, the use of AC and EFs together is better when faced with high-temperature and humidity.

No clear instructions were given regarding the temperatures at which to turn on AC or ideal room temperature settings. The distributed pamphlets suggested that AC should be used according to the situation; therefore, the participants possibly did not fully understand when AC should be used.

The participants also gave a relatively low number of correct answers regarding relationships between heat-related illness and chronic diseases. Some studies have reported that extreme heat can exacerbate chronic diseases such as diabetes, cardiovascular, respiratory, renal, and mental diseases [18,22,23,24]. Incorrect knowledge can exacerbate not only heat-related illness, but chronic diseases as well.

## 5. Limitation

Because almost 30% of the sampled subjects did not respond to the survey or dropped out in the trial, there is some concern of the selection bias, which could have resulted in higher effectiveness of the intervention than truth. Furthermore, elderly people tend to lose temperature sensibility due to aging, thus implying that some of them might not believe that they were not at risk in the heat. This might show better result than the actual intervention effect.

There may be concerns about potential cross contamination of the HHWs into the control group. However, in the area of the control group, audio terminals and optical network have not been installed. There are no announcement devices in public places; they have only been installed in each house of the participants in the HHW or HHW+W groups. Hence, even if the participants in the control group visited the HHW or HHW+W areas, they could not hear the HHWs unless they were inside the houses. Additionally, the participants were randomly selected based on the administrative areas defined by geographical clusters, and mountainous areas exist between boundaries of the groups. For this reason, we believe that cross contamination was unlikely to have occurred by the design.

A specific health outcome, such as morbidity or mortality, was not investigated in this study because the aim was to assess behavior changes in the elderly. To reduce the adverse effects of high temperatures and humidity on health, we believe that it is necessary to first evaluate whether HHWSs can be effective in changing behaviors because it has been reported that the mere availability of a HHWS does not necessarily lead to behavioral changes [10] and, in the absence of behavioral changes, it is almost impossible to determine the protective effect of an intervention on morbidity or mortality. In future studies, it may be desirable to include health outcomes to investigate reductions in the health burden as a result of the interventions.

## 6. Conclusions

Some evidence was found to show that the provision of HHWs improved behaviors to prevent heat-related illness in the elderly. The additional household water delivery improved some additional behaviors to prevent heat-related illness. The results indicate that an individual-based approach in addition to a community-based HHW may be needed to raise awareness. Further studies to investigate whether the behavior changes caused by the interventions can be linked to improved health outcomes are needed.

## Appendix

### A1. Definitions Used in this Study for the Behavior Improvement

#### 1. How many hours did you use AC in daytime (until sunset)?

(1) rarely (2) 1–2 hours (3) 3–4 hours (4) more than 5 hours.

##### Definition of Improvement Group

- Participants who used AC longer in the daytime in the follow-up questionnaires than in the baseline questionnaires.
- Participants who chose Option (4) in both the baseline and follow-up questionnaires. These participants had used AC more than five hours in daytime even before the intervention.

#### 2. How many hours did you use AC in nighttime (until sunrise)?

(1) rarely (2) 1–2 hours (3) 3–4 hours (4) more than 5 hours.

##### Definition of Improvement Group

- Participants who used AC longer in nighttime in the follow-up questionnaires than in the baseline questionnaires.
- Participants who chose Option (4) in both the baseline and follow-up questionnaires. These participants had used AC more than five hours in nighttime even before the intervention.

#### 3. At what temperature did you start using AC?

(1) less than 26 °C (2) 26–27 °C (3) 28–29 °C (4) 30 °C and higher (5) when feeling hot.

##### Definition of Improvement Group

- Participants who chose Options (3–5) in the baseline questionnaires and chose either Options (1) or (2) in the follow-up questionnaires. These participants started using AC at ≥28 °C or when feeling hot in the baseline, and started using AC at <28 °C after the intervention.
- Participants who chose either Option 1 or 2 in both the baseline and follow-up questionnaires. These participants had used AC at <28 °C even before the intervention.

#### 4. What were the room temperature settings?

(1) less than 26 °C (2) 26–27 °C (3) 28–29 °C (4) 30 °C and more (5) not decide.

##### Definition of Improvement Group

- Participants who chose Options (3–5) in the baseline questionnaires, and chose either Options (1) or (2) in the follow-up questionnaires. These participants set the room temperature to ≥28 °C or did not decide the temperature in the baseline, and set the room temperature to <28 °C after the intervention.
- Participants who chose either Options (1) or (2) in both the baseline and follow-up questionnaires, which means these participants had set the room temperature to < 28°C before the intervention.

#### 5. How many hours did you use an electric fan a day?

(1) rarely (2) 1–2 hours (3) 3–4 hours (4) more than 5 hours.

##### Definition of Improvement Group

- Participants who used an electric fan longer in the follow-up questionnaires than in the baseline questionnaires.
- Participants who chose Option (4) in both the baseline and follow-up questionnaires. These participants had used an electric fan for more than five hours even before the intervention.

#### 6. How did you use a fan?

(1) use a fan with AC simultaneously (2.) do not use a fan when using AC (3) use only a fan (4) other.

##### Definition of Improvement Group

- Participants who chose either Options (2) or (3) in the baseline questionnaires, and chose Option (1) in the follow-up questionnaires. These participants used either an electric fan or AC before the intervention, and grew to use both at the same time after the intervention.
- Participants who chose Option (1) in both the baseline and follow-up questionnaires. These participants had used an electric fan and AC at the same time even before the intervention

#### 7. How often did you drink alcohol during summer?

(1) never (2) rarely (3) sometimes (4) almost every day.

##### Definition of Improvement Group

- Participants who grew to refrain from alcohol in the follow-up questionnaires more than in the baseline questionnaires.
- Participants who chose Option (1) in both the baseline and follow-up questionnaires. These participants had refrained from alcohol even before the intervention

#### 8. What kinds of clothes did you wear?

(1) jumper or sweatshirt (2) long-sleeved clothing (3) short-sleeved clothing and short pants.

##### Definition of Improvement Group

- Participants who grew to wear lighter clothes in the follow-up questionnaires than in the baseline questionnaires.
- Participants who chose Option (3) in both the baseline and follow-up questionnaires. These participants had worn light clothes before the intervention.

#### 9. How often did you drink fluid (excluding liquids included in meals)?

(1) days when you never drank fluid
(2) days when you hardly drank fluid
(3) drink sometimes even if did not feel thirsty
(4) drink regularly even if not feeling thirsty.

##### Definition of Improvement Group

- Participants who grew to drink fluid more frequently in the follow-up questionnaires than in the baseline questionnaires.
- Participants who chose Option (4) in both the baseline and follow-up questionnaires. These participants had drunk fluid regularly even before the intervention.

#### 10. Did you cool your body when feeling hot?

(1) never (2) rarely (3) sometimes (4) almost every day.

##### Definition of Improvement Group

- Participants who grew to cool their bodies down when feeling hot in the follow-up questionnaires than in the baseline questionnaires.
- Participants who chose Option (4) in both the baseline and follow-up questionnaires. These participants had cooled their bodies when feeling hot even before the intervention.

#### 11. Did you take a rest when you were active (doing agriculture, fishery and walking)?

(1) never took a rest regularly
(2) rarely took a rest regularly
(3) sometimes took a rest regularly
(4) tried to take a rest regularly.

##### Definition of Improvement Group

- Participants who grew to take a rest more frequently in the follow-up questionnaires than in the baseline questionnaires.
- Participants who chose Option (4) in both the baseline and follow-up questionnaires. These participants had taken a rest regularly even before the intervention.

#### 12. Were you active (doing agriculture, fishery and walking) during the hottest period of days (10 am to 4 pm)?

(1) active every day
(2) active frequently (not every day)
(3) sometimes refrained from being active
(4) tried to refrain from being active.

##### Definition of Improvement Group

- Participants who grew to refrain from activities during the hottest period in the follow-up questionnaires than in the baseline questionnaires.
- Participants who chose Option (4) in both the baseline and follow-up questionnaires. These participants had refrained from activities during the hottest period even before the intervention.

#### 13. Did you use a hat or parasol when going outside?

(1) never (2) rarely (3) sometimes (4) every day.

##### Definition of Improvement Group

- Participants who grew to use a hat or parasol more frequently in the follow-up questionnaires than in the baseline questionnaires.
- Participants who chose Option (4) in both the baseline and follow-up questionnaires. These participants had used a hat or parasol every day even before the intervention.

**Table A1:** CONSORT 2010 checklist of information to include when reporting a randomized trial *.

Section/Topic	Item No.	Checklist Item	Reported on Page No.
Title and abstract			
	1a	Identification as a randomised trial in the title	1
	1b	Structured summary of trial design, methods, results, and conclusions (for specific guidance see CONSORT for abstracts)	1-2
Introduction			
Background and objectives	2a	Scientific background and explanation of rationale	2
	2b	Specific objectives or hypotheses	2
Methods			
Trial design	3a	Description of trial design (such as parallel, factorial) including allocation ratio	3
	3b	Important changes to methods after trial commencement (such as eligibility criteria), with reasons	N.A.
Participants	4a	Eligibility criteria for participants	3
	4b	Settings and locations where the data were collected	3/Figure 1
Interventions	5	The interventions for each group with sufficient details to allow replication, including how and when they were actually administered	4
Outcomes	6a	Completely defined pre-specified primary and secondary outcome measures, including how and when they were assessed	5
	6b	Any changes to trial outcomes after the trial commenced, with reasons	N.A.
Sample size	7a	How sample size was determined	4
	7b	When applicable, explanation of any interim analyses and stopping guidelines	N.A.
Randomisation:			
Sequence generation	8a	Method used to generate the random allocation sequence	4
	8b	Type of randomisation; details of any restriction (such as blocking and block size)	4
Allocation concealment mechanism	9	Mechanism used to implement the random allocation sequence (such as sequentially numbered containers), describing any steps taken to conceal the sequence until interventions were assigned	4
Implementation	10	Who generated the random allocation sequence, who enrolled participants, and who assigned participants to interventions	4
Blinding	11a	If done, who was blinded after assignment to interventions (for example, participants, care providers, those assessing outcomes) and how	N.A.
	11b	If relevant, description of the similarity of interventions	N.A.
Statistical methods	12a	Statistical methods used to compare groups for primary and secondary outcomes	5
	12b	Methods for additional analyses, such as subgroup analyses and adjusted analyses	5
Results			
Participant flow (a diagram is strongly recommended)	13a	For each group, the numbers of participants who were randomly assigned, received intended treatment, and were analysed for the primary outcome	Table 1/Figure 2
	13b	For each group, losses and exclusions after randomisation, together with reasons	Figure 2
Recruitment	14a	Dates defining the periods of recruitment and follow-up	4
	14b	Why the trial ended or was stopped	Figure 2
Baseline data	15	A table showing baseline demographic and clinical characteristics for each group	Table 1
Numbers analysed	16	For each group, number of participants (denominator) included in each analysis and whether the analysis was by original assigned groups	Table 1
Outcomes and estimation	17a	For each primary and secondary outcome, results for each group, and the estimated effect size and its precision (such as 95% confidence interval)	Table 2/Table 3
	17b	For binary outcomes, presentation of both absolute and relative effect sizes is recommended	N.A.
Ancillary analyses	18	Results of any other analyses performed, including subgroup analyses and adjusted analyses, distinguishing pre-specified from exploratory	Table 2/Table 3
Harms	19	All important harms or unintended effects in each group (for specific guidance see CONSORT for harms)	N.A.
Discussion			
Limitations	20	Trial limitations, addressing sources of potential bias, imprecision, and, if relevant, multiplicity of analyses	20
Generalisability	21	Generalisability (external validity, applicability) of the trial findings	19-20
	22	Interpretation consistent with results, balancing benefits and harms, and considering other relevant evidence	19-20
Other information			
Registration	23	Registration number and name of trial registry	N.A.
Protocol	24	Where the full trial protocol can be accessed, if available	N.A.
Funding	25	Sources of funding and other support (such as supply of drugs), role of funders	21

* We strongly recommend reading this statement in conjunction with the CONSORT 2010 Explanation and Elaboration for important clarifications on all the items. If relevant, we also recommend reading CONSORT extensions for cluster randomised trials, non-inferiority and equivalence trials, non-pharmacological treatments, herbal interventions, and pragmatic trials. Additional extensions are forthcoming: for those and for up to date references relevant to this checklist, see www.consort-statement.org.
